# Monitoring of Bicelles Spreading into Floating Lipid Bilayers

**DOI:** 10.1021/acs.langmuir.5c02620

**Published:** 2025-07-30

**Authors:** Justyna Bożek, Damian Dziubak, Arkadiusz Grempka, Slawomir Sek, Izabella Brand

**Affiliations:** 1 Carl von Ossietzky Universität Oldenburg, Institute of Chemistry, 26111 Oldenburg, Germany; 2 University of Warsaw, Faculty of Chemistry, Biological and Chemical Research Centre, Zwirki i Wigury 101, Warsaw 02-089, Poland; 3 Research Center for Neurosensory Sciences, 11233 Carl von Ossietzky Universität Oldenburg, D-26111 Oldenburg, Germany

## Abstract

Bicelles are hybrid, disk-shaped aggregates. Bicelles with a diameter of 11.8 ± 0.2 nm containing a long-chain lipid (1,2-dimyristoyl-*sn*-glycero-3-phosphocholine, DMPC) in their core and a short-chain, rim forming, lipid (1,2-diheptanoyl-*sn*-glycero-3-phosphocholine, DHPC) were prepared. A 100-fold dilution of the stock bicelle solution destabilizes the aggregate structure. Under this condition, the bicelles spread onto a β-thioglucose:6-mercaptohexanoic acid monolayer modifying Au(111) surface to form a free-standing floating DMPC bilayer while the DHPC molecules form micelles and diffuse into the electrolyte solution. Electrochemical impedance spectroscopy, electrochemically controlled quartz crystal microbalance, and polarization modulation infrared reflection absorption spectroscopy were applied to probe the macroscopic properties and potential-driven molecular scale changes in the floating DMPC bilayer. They are dependent on the sign of the membrane potential. The high values of the membrane resistance (ca. 2 MΩ cm^2^) indicate the formation of a compact, defect-free bilayer. At negative membrane potentials, the membrane resistance and tilt of the acyl chains (thickness of the membrane) change linearly as a function of potential, indicating that ion conduction occurs through the defect-free bilayer. A transition to positive membrane potentials leads to an abrupt decrease in the membrane resistance and tilting of acyl chains. These sudden reorientations lead to the formation of defects in the membrane structure as observed in electrochemical impedance spectroscopy experiments. The floating DMPC bilayer spread from bicelles provides ideal conditions to incorporate transmembrane proteins.

## Graphical abstract

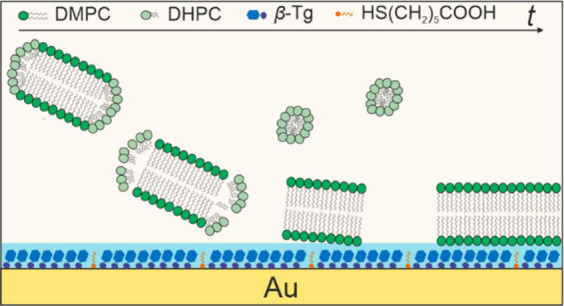

## Introduction

A biological cell membrane is a semipermeable fluid lipid bilayer with integrated or partially inserted proteins.^1,2^ It separates the inner and outer environments of a cell. Lipids and proteins present in this fluid membrane exhibit both vertical and transverse mobility that is essential to maintain specific functions of the membrane-associated proteins, locally change the membrane thickness and curvature, or initiate essential processes for cell life. The compositional complexity and permanent dynamic changes occurring in cell membranes make the studies of their structure and activity, as well as the elucidation of biological functions of individual components, very difficult. For these purposes, viable model systems are required. A single-component lipid bilayer is the simplest model of a biological cell membrane. Macroscopic properties such as membrane conductance, resistance, or capacitance, as well as the molecular-scale structure of multicomponent model membranes, which display lateral and transverse asymmetry, have been reported in the literature.^3–9^

Analytical studies of the structure and activity of the components of a model membrane usually require their deposition on a solid surface such as glass, mica, silicon oxide, or gold.^10–14^ In supported lipid bilayers the inner leaflet is deposited directly on a solid surface,^10,11,14^ introducing asymmetry in the orientation and hydration of the lipid molecules in both layers of a membrane.^9^ To improve the architecture of the model membrane, the introduction of an aqueous environment on both sides of the bilayer is required. Precoating of the substrate with a polymer of polyelectrolyte film provides a soft, hydrophilic environment that serves as a cushion for the deposition of a model membrane.^15–18^ Due to the roughness of polymer films, the lipid bilayer formed on their surfaces contained many defects.^16,17^ To improve the integrity of the model membranes, the use of small hydrophilic tether molecules was proposed.^19–21^ Use of lipid molecules in which a modified polar headgroup chemisorbs on a solid surface belongs to another strategy of the preparation of tethered lipid bilayers.^19,22^ Functionalized lipid molecules containing oligoethylene,^19^ ethylene oxide,^18,22,23^ oligopeptides,^24,25^ or ethoxy disulfide^22^ residues as a hydrophilic spacer and disulfide moiety chemisorbs on metallic surfaces were synthesized. The lipid bilayer is deposited either by vesicle spreading or Langmuir–Blodgett–Langmuir–Schaefer transfer. Alternatively, skeletonized surfaces were prepared to fabricate model lipid membranes that contact water on both sides.^26^ In this case, hydrophilic zirconium phosphonate or zirconium phosphate modified surfaces were used. The zirconated surfaces have a strong affinity for divalent phosphate. A strong binding was achieved by the addition of a low concentration of phosphatidic acid lipids to vesicles and Langmuir monolayers to prepare a model membrane. The lipid bilayers prepared using tether molecules or on skeletonized surfaces are characterized by labor-intensive fabrication protocols, which are specific to either selected surfaces or lipid molecules. The advantage is the presence of water on both sides of the membrane. However, the mobility of the lipid molecules is reduced due to binding of the lipid molecules with the molecules of the cushion layer.

Floating lipid bilayers offer an attractive approach for the fabrication of free-standing lipid bilayers separated by a water layer from the substrate surface.^27–30^ A floating model membrane is deposited either on a hydrophilic spacer: a self-assembled monolayer^27–30^ or on a supported lipid bilayer.^30^ A lipid membrane is deposited by spreading of vesicles or by Langmuir–Blodgett - Langmuir–Schaefer transfer. The supramolecular architecture of the floating bilayer is a result of attractive van der Waals interactions that keep the lamellar lipid bilayer structure and repulsive hydration forces that lift the membrane by 1–3 nm above the spacer surface.

In aqueous solutions, lipids form various aggregates whose shape and structure depend on the lipid geometry, water content, and temperature.^31^ Normal and inverted micelles, vesicles, hexagonal, lamellar or cubic phases are formed on single lipid–water mixtures. In tertiary mixtures containing a lipid with long hydrocarbon chains (e.g., 1,2-dimyristoyl-*sn*-glycero-3-phosphocholine, DMPC), a lipid with short hydrocarbon chains, called detergent (e.g., DHPC, diheptanoyl-*sn*-glycero-3-phosphocholine), and water, the phase diagram is enriched in hybrid aggregates such as bicelles.^6,32–36^ Bicelles are disk-shaped aggregates. The word *bicelle* reflects their composition: a *bi* layer of long-chain lipids forming the inner core and a mi*celle* of short-chain lipids (detergent) forming the rim of the aggregate. The size of the bicelle and the ratio between the diameter of the inner core and the diameter of the whole bicelle depend on the *q* value which is the molar ratio of long-chain to short-chain lipid and the level of hydration (*c*_L_, weight percent of total lipid mass to the total weight of the sample).^32,33^ Bicelles containing different long-chain lipids such as phosphatidylcholine, phosphatidylethanolamine, galactolipids, sphingolipids or cholesterol can be prepared for varying *q* and *c*_L_ values. As a short-chain (detergent) lipid, usually phosphatidylcholine was used. However, other molecules, such as 3-[(3-cholamidopropyl)-dimethylammonio]-2-hydroxy-1-propansulfonate were also used as detergent molecules that form a rim of bicelles.^23,34^ Dilution of the bicelles solution destabilizes the aggregate structure. Zeineldin et al. demonstrated for the first time that bicelles spread to form supported lipid bilayers on the silicon surface.^37^ In this pioneering study, the adsorption and spreading of bicelles yielding a 1,2-dipalmitoyl-*sn*-glycero-3-phosphocholine supported lipid bilayer was monitored by atomic force microscopy (AFM). Kolahdouzan et al. used quartz crystal microbalance and fluorescence microscopy to monitor the spreading of bicelles to supported bilayers on a silicon oxide surface.^38^ It was proposed that with the increase in the surface concentration of adsorbed bicelles, the short-chain phospholipids (dihexanoyl-*sn*-glycero-3-phosphocholine or diheptanoyl-*sn*-glycero-3-phosphocholine) solubilize into the solution phase, forming a well-ordered, defect-free lipid bilayer.^37–40^ Bicelles were also used to fabricate a tethered DMPC lipid bilayer on a Au(111) surface.^23^ For electrochemical characterization, the membrane resistance and capacitance indicated a formation of a compact, well-organized bilayer. Dziubak et al. also used bicelles to fabricate floating membranes on a gold surface modified by a β-thioglucose (β-Tg) monolayer.^41^ In this case, however, a double bilayer with numerous defects and pinholes was formed. After a freeze–thaw treatment, the assembled structure underwent morphological changes to yield a rather compact single bilayer.

In this work, we used a method previously described by Dong et al.^42^ to fabricate bicelles with an average diameter of 11.8 ± 0.2 nm. Dilution of the stock bicelles solution by a factor of 100 resulted in their spreading to form a DMPC bilayer on a β-Tg:6-mercaptohexanoic acid (HS­(CH_2_)_5_COOH, *abb*. SC_5_COOH) monolayer modified Au(111) surface. The two-component spacer monolayer is required to facilitate adsorption and uniform orientation of bicelles filled with transmembrane proteins, which is a subject of our future studies. Quartz crystal microbalance with energy dissipation (QCM-D) experiments confirmed mass change that corresponds to the formation of a single bilayer in the modified Au(111) electrode surface. The bicelles’ spreading was monitored by surface enhanced infrared reflection absorption spectroscopy (SEIRAS), which proved indeed that the long chain *d*_54_-DMPC (per-deuterated 1,2-dimyristoyl-*sn*-glycero-3-phosphocholine) lipid forms a bilayer while the short *h*_26_-DHPC (1,2-diheptanoyl-*sn*-glycero-3-phosphocholine, detergent) lipids form micelles and diffuse into the electrolyte solution. The electrochemical impedance spectroscopy (EIS) results indicate a formation of a compact, well-packed, defect-free bilayer whose resistance and capacitance are comparable to DMPC floating bilayer formed by Langmuir–Blodgett - Langmuir–Schaefer transfer.^43–46^ Change in the sign of the membrane potential triggers changes in the membrane structure and electrochemical properties. Polarization modulation infrared reflection absorption spectroscopy (PM IRRAS) experiments with electrochemical control gave a molecular-scale picture of potential-driven changes in the floating bilayer. Changes in the membrane resistance (or conductance) are accompanied by a continuous change in the tilt of the acyl chain and a restricted reorientation of the ester carbonyl group. Hydration and circulation of water/ions stiffen the polar headgroup region of the DMPC floating membrane, spread from bicelles. Spreading of bicelles is an alternative method for reproducible preparation of model membranes, in particular offering possibilities for an easy way of incorporation of transmembrane proteins into the model lipid bilayer.

## Experimental Section

### Chemicals

To fabricate bicelles, 1,2-dimyristoyl-*sn*-glycero-3-phosphocholine (DMPC, 14:0/14:0), per-deuterated *d*_54_-DMPC, and 1,2-diheptanoyl-*sn*-glycero-3-phosphocholine (DHPC) were purchased from Avanti Polar Lipids (USA). Deuterium oxide (D_2_O) was obtained from Deutero (Germany). 1-thio-β-d-glucose sodium salt, 6-mercaptohexanoic acid, potassium phosphate dibasic, potassium phosphate monobasic, hydrogen peroxide (30%, puriss. p.a.), and chloroform (99%) were from Sigma-Aldrich (Germany). Ethanol, methanol, and sulfuric acid (95%, puriss. p.a.) were from AnalaR Normapur, VWR (France). Potassium hydroxide was purchased from Merck/Sigma-Aldrich (Germany). All aqueous solutions were prepared using freshly filtered water with a conductivity of ≤ 0.50 μS cm^–1^ (PureLab Classic, Elga LabWater, Celle, Germany).

### Preparation of Bicelles

Bicelles were prepared at a total lipid concentration of 250 mM with a molar ratio *q* = 0.5, following the method previously described by Dong and co-workers.^42^ For a preparation of 0.4 mL of bicelles, lipids in powder form (22.59 mg of DMPC and 32.10 mg of DHPC) were first dissolved in phosphate buffer (concentrated K_2_HPO_4_/KH_2_PO_4_ (pH 7.2)) using 0.25 and 0.15 mL of buffer, respectively. The total lipid concentration *c*_L_ equals 12% (w/w). The resulting milky DMPC solution was vortexed and incubated in a thermomixer at 42 °C for 5 min. The DHPC solution was then added, gently mixed, and further incubated at 42 °C for 10 min. The final mixture, with a lipid molar ratio of *q* = 0.5 and total lipid concentration of 250 mM (corresponding to *c*_L_ 12% w/w), was then stirred at room temperature for 1 h until it became clear. The resulting bicelles remained stable for up to 48 h in the refrigerator and could be stored at −20 °C after freezing in liquid nitrogen.

### Spreading of Bicelles to a Floating Lipid Bilayer

A Au­(111) disc (3 mm diameter in electrochemical measurements and 15 mm diameter in spectroelectrochemical experiments, MaTeck, Germany) was cleaned using piranha etch (96% sulfuric acid:30% hydrogen peroxide, 3:1), thoroughly rinsed with water, and flame-annealed. The cleaned electrode was then immersed in a self-assembly solution containing 1.98 mM 1-thio-β-d-glucose (β-Tg) in water and 0.02 mM 6-mercaptohexanoic acid (SC_5_COOH) in methanol for at least 12 h. After self-assembly, the modified Au(111) surface was rinsed with water. Next, the electrode was placed in a bicelle solution diluted with the phosphate buffer solution (KH_2_PO_4_/K_2_HPO_4_ (pH 7.2)) to a total lipid concentration of 2.5 mM, incubated for 30 min, and gently rinsed. After drying for 1 to 3 h, the electrode was ready for measurements.

### Electrochemical Experiments

Electrochemical measurements were conducted using an AutoLab potentiostat in an all-glass, three-electrode cell with a hanging meniscus configuration. The working electrode (WE) was a Au(111) disk, while the counter electrode consisted of a 1 mm thick gold wire coiled into a ∼10 mm diameter flat disc. A saturated Ag|AgCl (KCl) electrode served as the reference electrode. All potentials defined below are referenced versus the RE. All experiments were carried out in a 50 mM phosphate buffer solution (KH_2_PO_4_/K_2_HPO_4_ (pH 7.2)). Cyclic voltammetry curves were recorded at a scan rate of 20 mV s^–1^, with a current range of 10 μA and a potential interval of 0.005 V. EIS was conducted over a frequency range of 10^5^ to 10^–2^ Hz, with 10 frequency points per decade and an amplitude of 0.005 V_rms_. Measurements were performed every −0.1 V in the potential range from 0.1 to −0.4 V in the negative-going scan and at −0.2, 0.1, and 0.2 V in the positive-going potential scan. Prior to each experiment, the electrochemical cell was deaerated by purging with argon for 30 min while the working electrode remained withdrawn from the solution to prevent damage to the lipid bilayer. During the measurements, argon flow was maintained above the solution to minimize oxygen interference. Impedance spectra were analyzed using ZSimpWin software (AMETEK Scientific Instruments, USA).

### PM IRRAS

PM IRRA spectra were recorded using a Vertex 70 spectrometer with a photoelastic modulator (*f* = 50 kHz; PMA 50, Bruker, Germany) and demodulator (Hinds Instruments, USA). A custom-made glass cell was cleaned using freshly prepared piranha etch (96% sulfuric acid:30% hydrogen peroxide, 3:1), thoroughly rinsed with water, and then dried overnight in an oven at 60 °C. The spectroelectrochemical cell represents the following stratified system of the passage of the IR beam: CaF_2_ optical window, electrolyte solution, model membrane assembly, Au electrode, and mirror of the IR radiation.^47^ The CaF_2_ prism was rinsed with ethanol, water, and ethanol, then dried under an argon stream and placed in a UV ozone chamber (Bioforce Nanosciences, USA) for 10 min. A disc polycrystalline gold electrode (15 mm diam., MaTeck, Germany) modified by a β-Tg:SC_5_COOH monolayer and a floating lipid bilayer prepared by bicelles spreading was used as the working electrode and mirror for the IR radiation. The counter electrode, a platinum ring, is built in the spectroelectrochemical cell. The reference electrode was Ag|AgCl in 3 M KCl in D_2_O. The electrolyte solution was a 50 mM phosphate buffer solution KH_2_PO_4_/K_2_HPO_4_ in D_2_O (pD 7.5) (the difference between pH and pD is approximately 0.4).^48^ The electrolyte solution was deaerated for 30 min by purging with argon. At each potential applied to the electrode during spectroelectrochemical measurements, 400 spectra at a resolution of 4 cm^–1^ were collected. For the analysis of the CH stretching modes the half-wave retardation was set to 2900 cm^–1^ and the angle of incidence of the IR radiation was 55°. For the analysis of the C=O and COO^–^ stretching modes the half-wave retardation was set to 1600 cm^–1^ and the angle of incidence of the IR radiation to 60°. The thickness of the electrolyte layer between the prism and the working electrode ranged between 3 and 8 μm. During the measurement, the following potentials were applied on the negative-going: 0.25, 0.2, 0.1, 0.0, −0.1, −0.2, −0.3, and −0.4 V and positive-going: −0.3, −0.2, −0.1, 0.0, 0.1, and 0.2 V potential scans. The analysis of the PM IRRA spectra was carried out using the OPUS v 5.5 software (Bruker, Germany).

### SEIRAS

Lipid bilayer formation from bicelles on a gold surface was monitored using SEIRAS. The SEIRAS spectra were recorded with a Nicolet iS50 FTIR spectrometer (Thermo Scientific, USA) equipped with an MCT-A detector and a custom-designed single reflection accessory. The spectral resolution was set at 4 cm^–1^, with an incident angle of 60°. The experimental setup closely resembled that of attenuated total reflection (ATR) spectroscopy; however, in this configuration, a hemisphere prism was covered with a thin layer of gold, as described previously.^49^ Briefly, the surface of a hemispherical silicon prism was etched with 2% HF solution. Then, the prism was washed thoroughly with Milli-Q water. Deposition of gold was performed by placing an aqueous plating solution (mixture of 100 μL of 0.03 mol L^–1^ NaAuCl_4_ × 2H_2_O, 2 mL of 0.15 mol L^–1^ Na_2_SO_3_ + 0.05 mol L^–1^ Na_2_S_2_O_3_ × 5H_2_O+ 0.05 M NH_4_Cl, and 1 mL of 2% HF) onto the prism’s surface. The deposition process was terminated by immersing the prism in water once a thin gold layer formed. Then, the prism was dried and prepared for further use. A β-Tg:SC_5_COOH (98:2 mol ratio) monolayer was self-assembled on the gold surface of the prism by immersing it in a mixture of this solution overnight. All spectroscopic experiments were performed in the phosphate buffer (pD 7.6) in D_2_O. To distinguish the CH_2_ vibrational bands of the DHPC and DMPC lipids, bicelles containing *d*_54_-DMPC and *h*_26_-DHPC were used. The spectra are presented as an absorbance versus wavenumber, where Δ*A* is defined as a −log­(*I*/*I*_0_), *I*_0_ is a background spectrum measured for the gold layer modified with β-Tg:SC_5_COOH monolayer in phosphate buffer prepared in D_2_O, while *I* is a measured spectrum after addition of the suspension of bicelles.

### QCM-D

Quartz crystal microbalance with energy dissipation (Q- Sense E1 instrument, Q-Sense AB, Sweden) and a dedicated electrochemical cell (Electrochemistry Module, Q-Sense AB, Sweden) were used for the measurements. A miniature Ag|AgCl electrode (saturated with KCl, Q-Sense, Sweden) was employed as a reference electrode. The platinum ring in the cell was used as a counter electrode, while the gold QCM (QSX 338) sensor as a working electrode. The applied potential was controlled by a CHI 750E bipotentiostat (CH Instruments Inc., USA). During the experiments, the cyclic voltammetry was recorded for 5 consecutive scans. The potential was scanned from the positive value of +0.1 V (vs Ag|AgCl) to the negative value of −0.4 V (vs Ag|AgCl) at a scan rate of 1 mV s^–1^.

### Atomic Force Microscopy

Prior to the AFM measurements, a Au(111) disc was cleaned with freshly prepared piranha solution (sulfuric acid 96%:hydrogen peroxide 30%, 3:1) for 24 h; then, it was flame annealed. The gold substrate was left in an aqueous solution of β-Tg:SC_5_COOH (98:2 mol ratio) for self-assembly for 20h. After the self-assembly, the substrate was dried and mounted on the AFM sample stage. The AFM images and force-curves were recorded using a Dimension Icon Atomic Force Microscope (Bruker; Germany) in a liquid column of a 50 mM phosphate buffer solution, KH_2_PO_4_/K_2_HPO_4_ created between the substrate and the AFM holder. ScanAsyst-Fluid+ tips (Bruker, Germany) were used for imaging, with a resonance frequency of 150 kHz, a force constant of 0.7 N m^–1^, and a tip radius of 2–12 nm. Imaging was performed in PeakForce QNM mode, which, in addition to topographical measurements, enables the assessment of nanomechanical properties such as Young’s modulus, based on the Derjaguin, Muller, and Toporov (DMT) adhesion theory.^50,51^ Before the imaging, the tips were calibrated to obtain the accurate value of the force constant. First, images of the gold with the self-assembled monolayer of β-Tg:SC_5_COOH were recorded. Then, the bicelle solution (2.5 mM total lipid concentration) was added to the liquid column and allowed to spread on the substrate for a specified duration of 1h, and the images were recorded again. Additionally, force–distance ramps were collected every 100 nm across a 15 × 15 grid. The QNM images were analyzed using Gwyddion software, while force–distance ramps were processed using a custom Python script and visualized with Origin software.

## Results

### Observation of the Bicelles Spreading to a Floating Bilayer

The spreading of bicelles to supported lipid bilayers on silica and glass surfaces was described based on experiments utilizing quartz crystal microbalance with dissipation monitoring (QCM-D).^39,40,52^ The formation of a lipid bilayer results in a decrease in frequency to ca. −28 Hz.^40,53^ Therefore, we used QCM-D as a reference to confirm that DMPC:DHPC (*q* = 0.5) bicelles spread to yield a single lipid bilayer floating above the β-Tg:SC_5_COOH monolayer modifying the gold surface. Figure S1 shows the frequency versus time plots recorded over 30 min of the bicelles spreading. Bicelles solution (2.5 mM) was injected over the β-Tg:SC_5_COOH monolayer adsorbed on a gold sensor (100 nm thickness of the gold layer, < 1 nm RMS) modified quartz sensor (arrow 1 in Figure S1). The adsorption of bicelles caused an appearance of a deep minimum in frequency which finally, after washing of the sensor surface with the phosphate buffer (arrow 2 in Figure S1), led to a frequency decrease to −28 ± 1 Hz, a value that is characteristic for spreading of a lipid bilayer on a solid surface.^38–40,53^

According to the literature, during the formation of a lipid bilayer on a solid support, short-chain lipids are detached and diffuse into the bulk of the solution as a result of the fusion of bicelles.^2–4^ To follow *in situ* the structural changes in the bicelle architecture during their spreading to a bilayer SEIRA spectroscopy experiment was done. The bicelles were fabricated from *d*_54_-DMPC and *h*_26_-DHPC lipids. The isotope substitution shifts the CD stretching IR absorption bands in the *d*_54_-DMPC to the 2000–2200 cm^–1^ region, while the CH bands in DHPC appear in the 2800–3000 cm^–1^ spectral region. The results are shown in Figure 1

**Figure 1:**
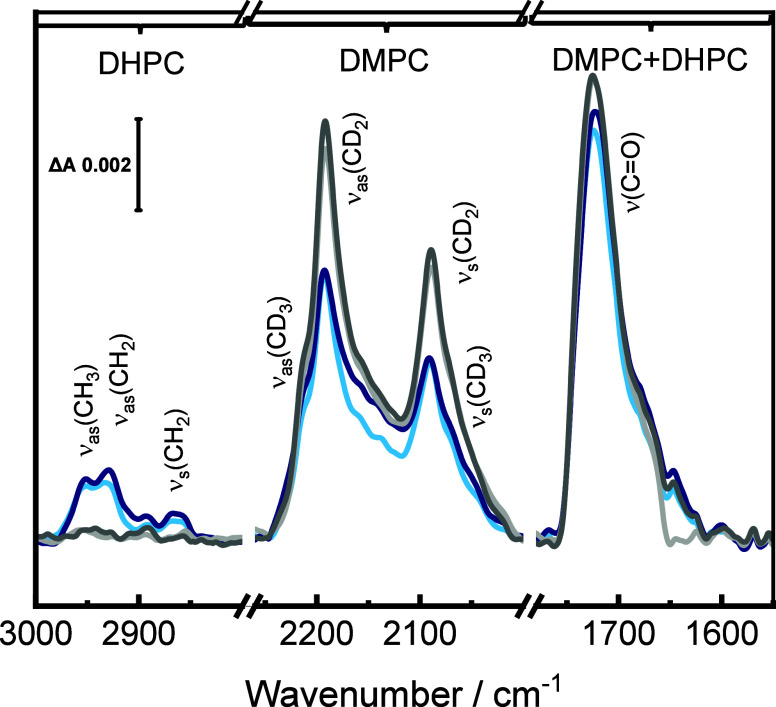
SEIRAS spectra showing the process of lipid bilayer formation from bicelles on a silicon prism covered by a 20 nm thick gold layer and modified by a β-Tg:SC_5_COOH monolayer in phosphate buffer prepared in D_2_O (pD 7.6). The blue lines represent the spectra recorded during the bicelle spreading: light blue for 30 min and navy for 60 min. The light gray line shows the spectrum after washing the cell with a fresh portion of phosphate buffer, immediately after rinsing, and the dark gray line the spectrum 30 min later.

Upon the addition of the bicelles suspension to the solution, new IR absorption bands appeared in the SEIRA spectra (blue lines in Figure 1). The weak IR absorption bands at 2952, 2927, 2891, and 2957 cm^–1^ correspond to the ν_as_CH_3_, ν_as_CH_2,_ ν_s_CH_3,_ and ν_s_CH_2_ in *h*_26_-DHPC, respectively.^5^ The stronger IR absorption bands in the 2220–2050 cm^–1^ arise from the CD stretching modes in *d*_54_-DMPC. The ν_as_CD_2_ and ν_s_CD_2_ modes appear at 2191 and 2092 cm^–1^ and correspond to a liquid ordered state of the hydrocarbon chains of the lipid molecules.^54,55^ The terminal methyl groups give the ν_as_CD_3_ band at 2216 cm^–1^ and the ν_s_CD_3_ at 2052 cm^–1^.^55,56^ The IR absorption mode centered at 1725 cm^–1^ arises from the ν­(C=O) of the ester carbonyl group in the lipid molecules.^57^ The SEIRA spectra measured after 30 and 60 min of bicelles spreading show no significant differences, indicating that the formed floating bilayer is stable over time. Note that the intensities of the CH stretching vibration bands showed no significant changes over the bilayer spreading time, indicating that the DHPC molecules are present either in the membrane or in its vicinity.^38–40^ Only after exchange of the buffer solution, the signals from the CH stretching modes disappeared from the spectra (Figure 1 (gray lines)). This result proves that DHPC molecules do not contribute to the membrane structure. After washing, the intensities of the CD stretching modes in *d*_54_-DMPC increased while the intensity of the ν­(C=O) absorption band remained practically unchanged (Figure 1). This behavior points to some reorientations of the lipid molecules in the floating bilayer caused by the buffer exchange. An increase in the intensities of the CD stretching modes indicates an increased, with respect to the surface normal, tilt of the acyl chains.^58–60^ A minor increase in the intensity of the ν­(C=O) mode (Figure 1) indicates compensation of the intensity loss due to removal of DHPC by the molecular scale rearrangements in the floating *d*_54_-DMPC bilayer.

The process of bicelles spreading to a floating membrane was visualized using AFM. Figure S2 shows AFM topography images of the floating DMPC bilayer spread from bicelles. After 30 min of the bicelles spreading, a floating membrane is clearly visible in the AFM images. The lipid membrane covers almost uniformly the β-Tg:SC_5_COOH monolayer modified Au(111) surface. The surface coverage calculated from Figure S2 equals 90%, indicating the formation of a compact floating bilayer with only a few defects. Based on Peak-force QNM images, the elastic modulus (Young’s modulus) for the gold substrate modified with a β-Tg:SC_5_COOH monolayer and a DMPC floating bilayer, obtained after the spreading of DMPC bicelles, was determined (Figure S3). The value of the Young’s modulus for β-Tg:SC_5_COOH monolayer is 7.2 ± 1.2 GPa. For example, decanethiol self-assembled monolayers are characterized by a Young modulus of ∼1 GPa^61^ while the Young modulus of oligourea monolayers was in the range of 0.7–1.1 GPa.^62^ This high value stems from the rigid substrate (gold) beneath a thin, soft monolayer. After the spreading of bicelles to a floating DMPC bilayer, the Young modulus decreased to 44.0 ± 2.8 MPa (Figure S3). The obtained value falls within the broad range of Young’s modulus values reported in the literature.^63–66^ For gel phase membranes it spans from tens to hundreds of MPa. For example, Et-Thakafy et al. reported Young’s modulus values of 10–20 MPa for gel-phase supported lipid bilayers, and 4–6 MPa for fluid-phase bilayers, based on AFM indentation measurements on mica-supported membranes.^67^ Furthermore, bilayers composed of dioleoylphosphatidylcholine:egg sphingomyelin:cholesterol, both in the absence and presence of ceramide, exhibit modulus values reaching the order of hundreds of MPa.^68^

Additionally, over the floating bilayer surface shown in Figure S2, force–distance curves were acquired. From 225 measured curves, 78 approach curves with a straight baseline, steep incline near the distance of 0 nm, and the presence of a characteristic rupture point were chosen for quantitative analyses. They are plotted in Figure 2A. Initially, the tip is far from the sample so that no deflection is observed (zero force). As the tip comes into contact with the membrane, the force starts to increase until coming to a lipid bilayer breakthrough point (Figure 2A). The approach curve, after reaching of the breakthrough point, shows a decrease in force (attraction of the tip within the spacer layer), followed by a steep sharp increase of the repulsive force when the tip reaches the gold surface (substrate). The breakthrough force (*F*_p_), statistically determined, equals 1.94 ± 0.20 nN (Figure 2B), while the thickness of the compressed membrane at this point *d*_0_ = 3.51 ± 0.64 nm (Figure 2A,C). The thickness of the compressed floating DMPC bilayer is smaller than 4.5–5.6 nm.^69–71^ Considering that the bilayer is separated from the gold surface by a ca. 1–3 nm thick water layer^29^ and a β-Tg:SC_5_COOH monolayer, the expected thickness of the floating membrane is in the range 6–9 nm. The breakthrough distance corresponds to the thickness of the compressed (deformed) membrane and therefore is smaller than the real thickness of lipid bilayers.^27,66^

**Figure 2:**
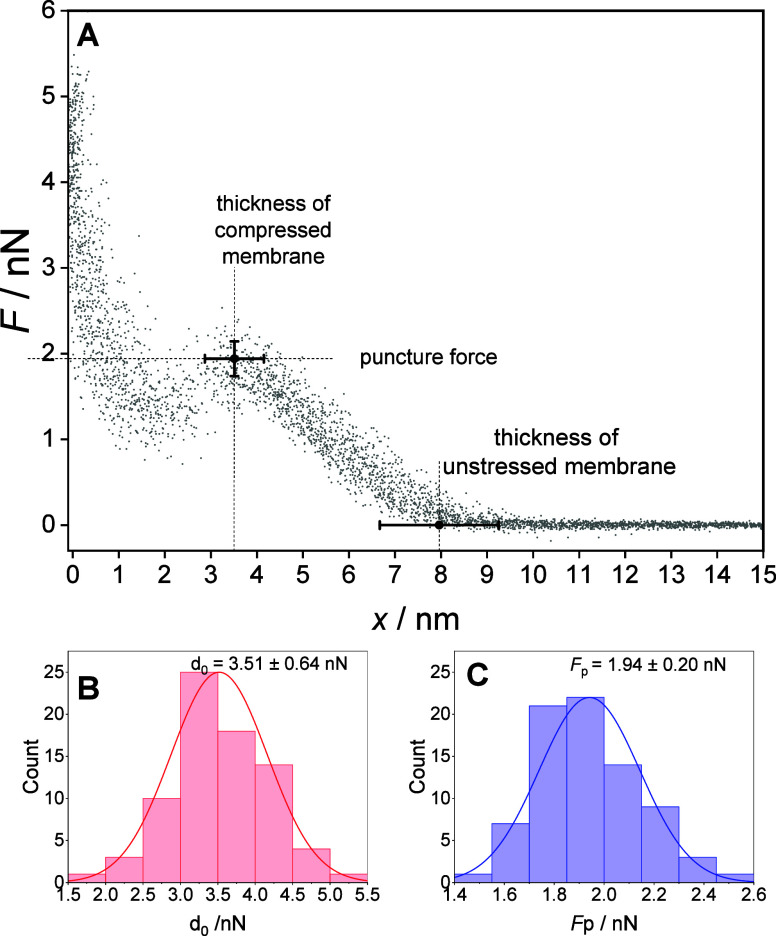
(A) Plot representing 78 force–distance curves (extended curves) altogether. Two points are marked. One represents the mean puncture force and distance (thickness of the compressed membrane), and the other the thickness of the unstressed membrane calculated from the Hertz model. (B) Histograms of the thickness of the compressed membrane, *d*_0_. (C) Histograms of the puncture force.

The total thickness of the floating bilayer can be determined from the force–distance curves between the onset compression point and the contact point with Au(111) surface (Figure 2A). A sum of the thickness of the compressed and the elastic deformation (indentation) gives the thickness of the unstressed membrane. The elastic deformation (indentation depth) (*δ*_*0*_) produced by the AFM tip under the load force can be determined from the Hertzian contact model,^72^

1 $$\delta_{0}=\sqrt[{3}]{\frac{0.75^{2}{\left(1-\mu^{2}\right)}^{2}F_{p}^{2}}{RE^{2}}}$$

where μ is the Poisson ratio (in the membrane assumed its limit value of 0.5), *R* is the radius of the AFM tip, *E* is the effective elastic modulus, and *F*_p_ is the breakthrough force.^27,73^ Using the *F*_*p*_ = 1.94 nN, the elastic modulus based on QNM measurements (44.0 ± 2.8 MPa) and the tip radius of 7 ± 5 nm, the calculated indentation depth δ_0_ = 4.45 ± 1.12 nm. Thus, the total thickness of the unstressed floating membrane formed by bicelles spreading equals 7.96 ± 1.29 nm and agrees with the expected thickness of the floating membrane. For example, Kycia determined the thickness of floating lipid membranes containing DMPC:GM1:cholesterol to range between 7.2 and 9.3 nm.^27^ The thickness of a floating POPC–POPS bilayer on a carboxylic acid-terminated thiooligoethylene glycol spacer molecule was 8.1 nm.^74^

### Stability of a Floating Bilayer in Electric Fields: EIS

EIS was used to test the electrochemical stability of the floating DMPC bilayer obtained by spreading of bicelles. The potential of zero free charge, *E*_pzfc_ of the floating DMPC bilayer deposited on a β-Tg:SC_5_COOH monolayer, equals 0.14 V versus Ag|AgCl reference electrode (Figure S4). The difference *E*-*E*_pzfc_ is a good approximation of the membrane potential, *E*_m_.^75^

Figure 3 shows the absolute impedance and phase angle spectra of the bilayer in 50 mM phosphate buffer electrolyte solution of pH 7.2 in the negative-going (filled squares) and positive-going (open squares) potential scans. At negative membrane potentials (*E* ≤ 0.1 V) the phase angle displays a plateau of ∼86° at frequencies 0.01< *f* < 100, indicating that the impedance is dominated by the capacitance of the floating membrane (Figure 3B). The number of defects in the floating DMPC bilayer is negligible. No electric potential (membrane potential) driven electroporation of the floating bilayer was observed. Only a transition to positive membrane potentials, at *E* = 0.2 V, the phase angle values drop with a decrease in frequency (Figure 3B), pointing to the formation of potential-driven defects in the floating bilayer.^41,76–79^ Thus, the electrochemical characterization of bilayers assembled from bicelles suggests the formation of a compact, defect-free membrane.

**Figure 3:**
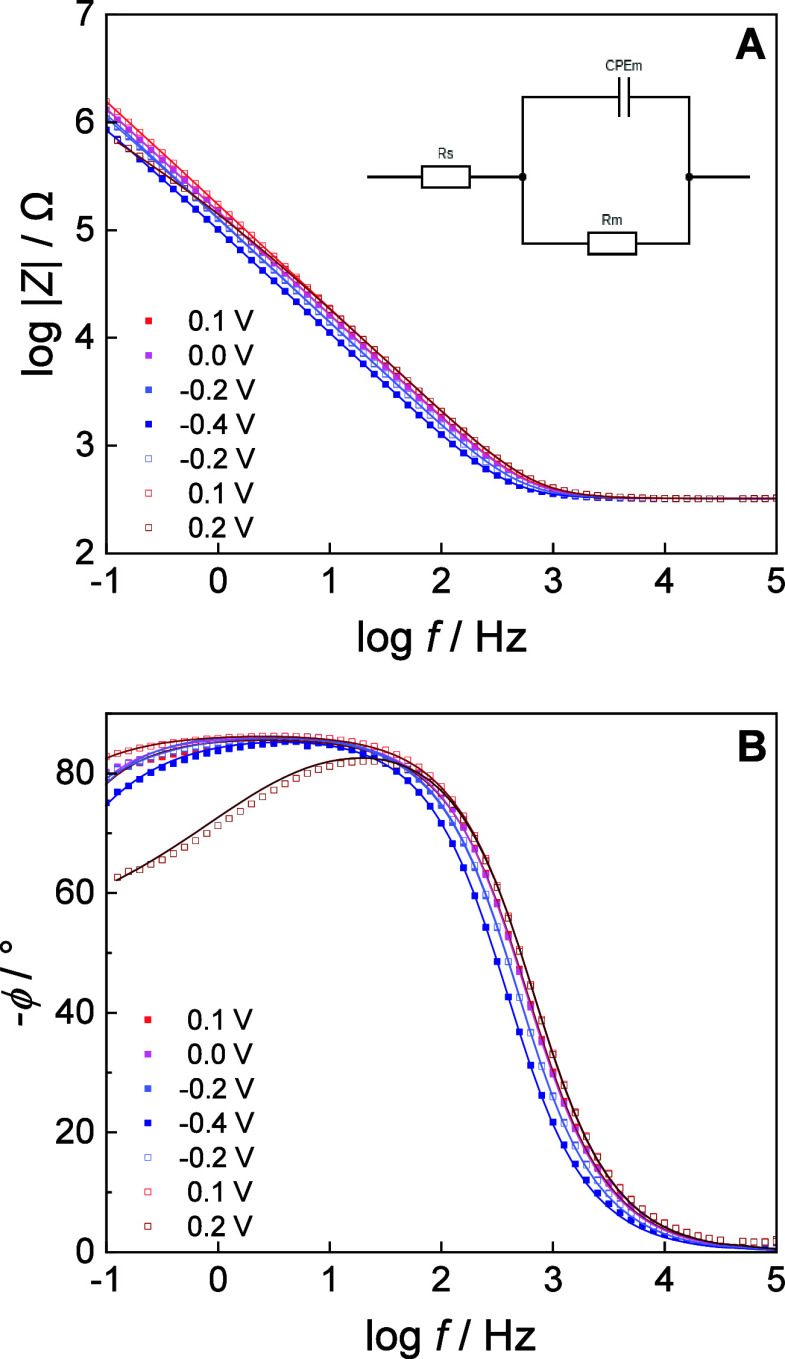
EIS results. (A) Absolute magnitude of impedance vs frequency and (B) phase angle vs frequency plots of a floating DMPC lipid bilayer formed from bicelles on a β-Tg:SC_5_COOH modified Au(111) surface in 50 mM phosphate buffer in H_2_O (pH 7.2) at potentials indicated in the figure in a negative-going (filled symbols) and positive-going (empty symbols) potential scans. Squares represent the measured data, and the solid lines correspond to the fits obtained by the equivalent circuit. The inset in panel A shows the electrical circuit used to fit the measured data, which was drawn using https://www.circuit-diagram.org/editor.

An equivalent circuit, shown in the inset of Figure 3A, was fitted to the experimental data. In this model, *R*_s_ represents the resistance of the electrolyte solution, *R*_m_ and CPE_m_ the resistance and constant phase element of the environment of the floating bilayer, including the spacer layer. The α value varies between 0 and 1.^79,80^ The results of the numerical analysis of the EIS data are shown in Figure 4 and summarized in table S1.

**Figure 4:**
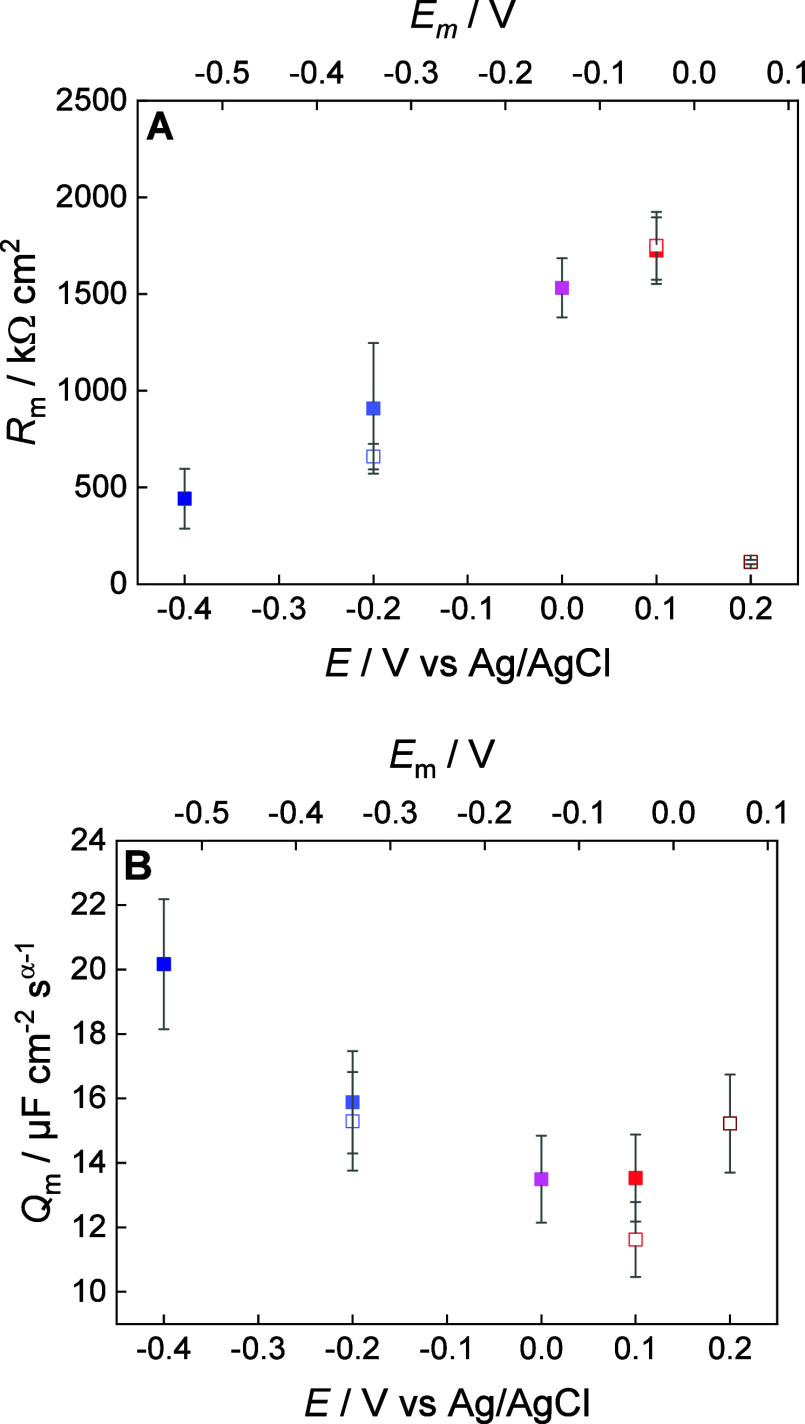
(A) Resistance (*R*_m_) and (B) constant phase element (*Q*_m_) of the floating bilayer on a β-Tg:SC_5_COOH monolayer modified Au(111) vs potential plots in 50 mM phosphate buffer in H_2_O (pH 7.2) in negative-going (blue squares) and positive-going (red squares) potential scans. Colors of the points correspond to potential values at which EIS measurements shown in Figure 3 were taken.

The *R*_m_ provides information about the membrane permeability to ions and water. At negative membrane potentials, both in the negative- and positive-going potential scans, the resistance (*R*_m_) of the floating bilayer changes linearly with potential reaching a maximum of 1.7 MΩ cm^2^ at *E* = 0.1 V (Figure 4A). The *R*_m_ values measured in our experiments are in the range of 0.2–8.0 MΩ cm^2^, reported for supported, tethered and floating lipid bilayers.^41,44,45,81^ A linear decrease in *R*_m_ (increase in the membrane conductance) in the negative-going potential scan (at negative membrane potentials) points to either alterations in the membrane structure or ion-membrane interactions and ion conduction. Since the EIS results show no electroporation process in the studied potential range (i.e., decrease in the phase angle at low frequencies), the ion conduction seems to be the primary process responsible for the changes in membrane resistance (conductivity). At low membrane potentials, when *R*_m_ is the highest, the ion transport through the hydrophobic membrane may involve the formation of ion pairs.^82^ As the net membrane potential increases, the degree of ion association decreases, which results in an increase in the membrane conductance. Interestingly, when the membrane potentials become positive (*E* = 0.2 V), a sudden drop in *R*_m_ to 0.12 MΩ cm^2^ is observed (Figure 4A). A decrease in phase angle (Figure 3B) indicates the formation of defects, which may indeed be responsible for increasing membrane ion conductivity.

EIS numerical analysis shows that the average α_m_ value equals 0.97 ± 0.01 (Table S1) and confirms that *Q*_m_ represents the capacitance of the membrane. The *Q*_m_ values of the floating DMPC bilayer depend on the membrane (electrode) potential as shown in Figure 4B. The *Q*_m_ minimum of 11 μC cm^–2^ appears in the vicinity of the *E*_m_ ≈ 0.0 V and increases by moving to negative and positive membrane potentials. The determined *Q*_m_ values are high compared to the capacitance of a defect-free lipid bilayer (∼1.0 μF cm^2^).^77,83^ Note that in the used equivalent circuit, the DMPC lipid bilayer and the spacer layer (water trapped between the lipid bilayer and the β-Tg:SC_5_COOH monolayer) contribute to the measured capacitance. The spacer layer contains components whose dielectric constants (water 78, glucose ca. 5–10 ^84^) are higher than the dielectric constant of the hydrocarbon chains (ca. 2) and lipid bilayers (e.g., egg lecithin bilayer 2.3–2.8 ^85^) explaining higher values of the capacitance of the floating membrane assembly. The analysis of the EIS results of a DMPC bilayer spread from bicelles gives a picture of a defect-free lipid bilayer, floating above a hydrophilic spacer layer. The negative polarization does not cause membrane electroporation, whereas switching to positive membrane potentials impacts the resistive (conductive) properties of the bilayer. The EIS results did not provide information about the spacer layer. To check the dynamic behavior of the floating membrane assembly, electrochemically controlled quartz crystal microbalance (QCM-D) experiments were performed. Figure 5 shows changes in the measured frequency and dissipation energy correlated to the potential applied to the Au(111) electrode as a function of time (number of potential scans). An extended graph of frequency changes over five potential cycles is shown in Figure S5. In the potential range between *E* ≤ −0.3 V in the negative- and *E* ≤ −0.2 V in the following positive-going potential scans, a maximum in the frequency (Figure 5A) and a shallow minimum in the dissipation energy (Figure 5B) were observed. The frequency changes by ∼1 Hz while the dissipation energy by ∼−0.1 ppm.

**Figure 5:**
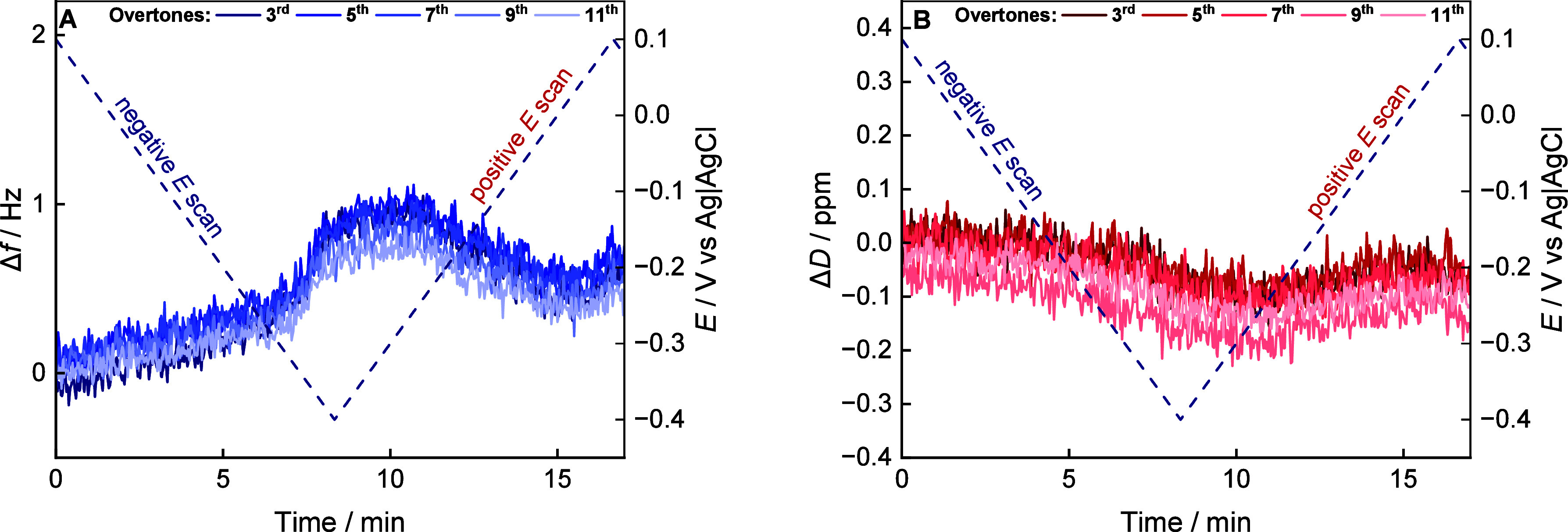
(A) Overtones of frequency and (B) dissipation energy of a QCM-D with a Au chip with a floating DMPC bilayer deposited by spreading of bicelles on a β-Tg:SC_5_COOH monolayer and changes in the potential applied to the Au electrode as a function of time, recorded during the first negative and following positive and negative potential scans. The potential scan rate was 1 mV s^–1^. The electrolyte solution contained 50 mM phosphate buffer in H_2_O (pH 7.2).

The appearance of a frequency maximum indicates a reduction in mass at negative potentials is compensated by an increase in mass of the membrane environment at positive potentials. In the potential range of the frequency maximum, dissipation energy exhibits a minimum, indicating that the decrease in mass at negative potentials is connected with a stiffening of the film in the vicinity of the electrode, followed by an increase in membrane elasticity at positive potentials. Taken together, the QCM-D experiments indicate a potential-driven circulation of water/ions from the spacer layer at the negative potentials when the *R*_m_ decreases (Figure 4A).

Over in six potential cycles an overall decrease in frequency by ca. 2.5 Hz for the third and 1.8 Hz for the 11th overtone was observed. It may be due to a loss of lipids from the bilayer (instability of the membrane itself), loss of components of the spacer layer in the following potential scans, or potential-dependent transport of hydrated ions in the electric field of the electrode. To prove the stability and structural integrity of the membrane, over eight potential scans of *in situ* PM IRRAS experiments were conducted.

### Structure of the Floating Bilayer Spread from Bicelles: *In Situ* PM IRRAS

Figure 6 presents the PM IRRA spectra in the CH stretching region of the acyl chains for the DMPC floating bilayer formed from bicelles, along with the spectrum of randomly distributed DMPC molecules in a bilayer-thick film. The spectra were collected over six potential cycles. No significant difference was observed in the intensities of the CH stretching absorption band in the first and last potential cycle, indicating that the floating DMPC bilayer is stable within the studied potential range (−0.55 V < *E*_m_ < 0.10 V).

**Figure 6:**
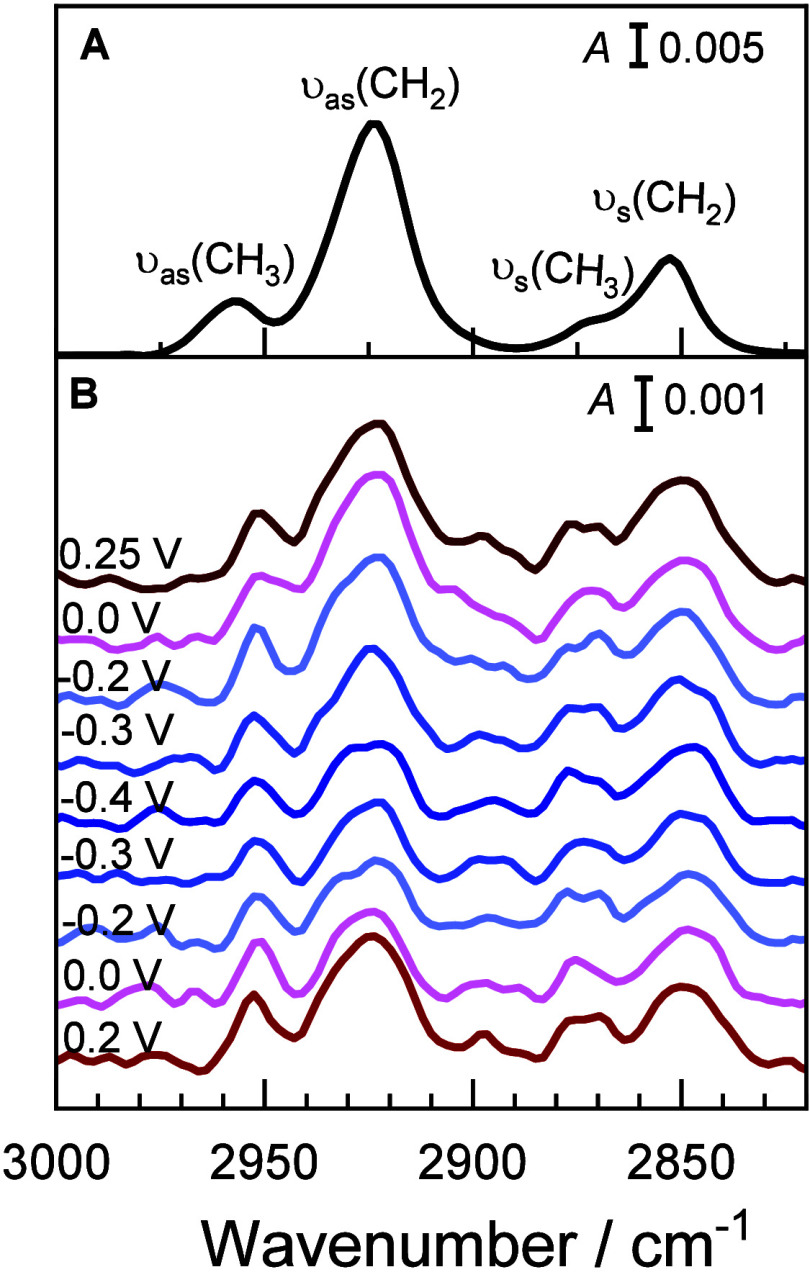
(A) PM IRRA spectrum of randomly distributed DMPC molecules in a bilayer thick film obtained from optical constants and (B) PM IRRA spectra of a floating DMPC bilayer obtained by spreading of bicelles on a β-Tg:SC_5_COOH monolayer adsorbed in a Au(111) electrode in 50 mM phosphate buffer in D_2_O (pD 7.6) at potentials marked in the figure in negative- and positive-going potential scans.

The hydrocarbon chains in DMPC exhibit four IR fundamental absorption bands: ν_as_(CH_3_), ν_as_(CH_2_), ν_s_(CH_3_), and ν_s_(CH_2_). In the *in situ* measured PM IRRA spectra, compared to the random spectrum, the methylene stretching IR absorption bands (12 per acyl chain) are weak, and comparable with the intensities of the methyl stretching (one per acyl chain) bands (Figure 6 and Figure S6).

The attenuation of the ν­(CH_2_) IR absorption modes in the floating bilayer is the consequence of the surface selection rule of IRRAS^86^ and a long-range order of the lipid molecules in the membrane.^43,46,47^ A negative potential shift leads to a weakening of the ν­(CH_2_) absorption bands, which increase again in intensity only in the following positive-going potential scan at *E* = 0.2, thus at positive membrane potentials (at *E*_m_ > 0 V). The observed changes are reversible versus the applied potential, indicating that in an organized molecular film such as a lipid bilayer, the tilt of the acyl chains in DMPC molecules in the membrane depends on the membrane (electrode) potential. The PM IRRA spectra were deconvoluted (Figure S6). The deconvolution results show that the positions of the CH_2_ stretching modes are independent of the electrode potentials and equal 2922.5 ± 0.6 cm^–1^ for the ν_as_(CH_2_) and 2849.0 ± 1.0 cm^–1^ for the ν_s_(CH_2_) band. The positions of these methylene stretching modes reflect the physical state of a hydrocarbon chain.^87^ In the floating DMPC bilayer obtained by spreading of bicelles, the acyl chains exist in a gel phase, indicating that the hydrocarbon chains in the lipid molecules adopt predominantly an extended all-*trans* conformation.^46^ This result is in agreement with rather high values, characteristic of a gel phase, of the Young modulus determined for the DMPC bilayer spread from bicelles.

The integral intensities of the methylene stretching modes were used to calculate the average tilt of the *trans* fragments of the acyl chains in the floating DMPC bilayer, as described in.^88^ Figure 7 shows the changes in the average tilt of the *trans* fragments of the acyl chains in the DMPC floating bilayer as a function of the electrode potential. During the negative-going potential scan (filled squares) the tilt of the acyl chains changes linearly with a slope of 18.5°/V (inset of Figure 7). At *E* = 0.25 V the tilt of the acyl is 35° versus surface normal, while at *E* = −0.4 V it decreases to 23°. Thus, the membrane thickness decreases continuously with increasing negative membrane potentials. A linear dependence of molecular-scale deformations of lipid bilayers as a function of the applied voltage has already been observed.^82,89,90^ A voltage-induced change in the arrangement, conformation, packing, and ordering of the lipid molecules may be responsible for changes of the membrane conductivity (resistance).^82,89–92^ The potential-dependent changes may include a rearrangement of the polar head groups in such a way that the lipid dipoles turn by ca. 1° per 0.1 V.^82,89,90^ The rearrangement of the polar head groups may induce rearrangements of the acyl chains. It was also shown that electric potentials (fields) may affect not only the tilt of the acyl chains but also cause their melting, increasing the number of gauche conformations.^46,93^

**Figure 7:**
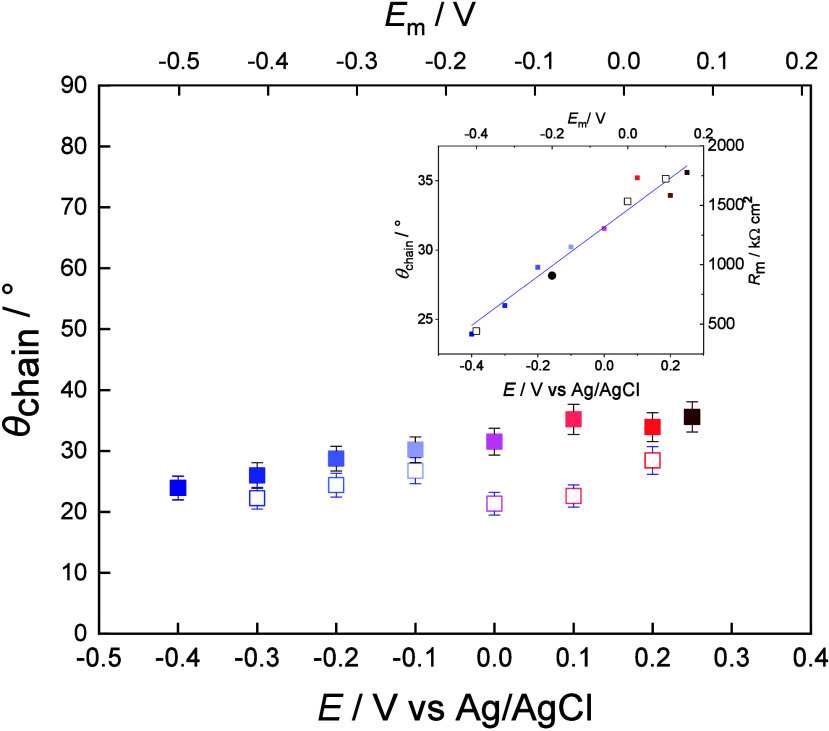
Tilt angle of the *trans* fragments of the acyl chains in the DMPC floating bilayer deposited on the β-Tg:SC_5_COOH monolayer on a Au(111) electrode surface vs potential (membrane potential) plots. The electrolyte solution contained 50 mM phosphate buffer in D_2_O (pD 7.6): negative-going (filled squares) and positive-going (empty squares) potential scans. Colors of the points correspond to potential values at which PM IRRA spectra were recorded, as shown in Figure 6. The inset shows the tilt angle (filled symbols) and *R*_m_ (empty symbols) vs potential plots of the DMPC floating bilayer in a negative-going potential scan.

The inset in Figure 7 shows that in the negative-going potential scan, the change in the tilt of the acyl chains corresponds to the changes in the membrane resistance (*R*_m_). The membrane resistance is highest at positive potentials, when the acyl chains have the largest inclination (35° vs surface normal) and the membrane has the lowest thickness. This result appears counterintuitive, since *R*_m_ tends to decrease with a decrease in the thickness of a blocking film. However, the high values of *R*_m_ (MΩ cm^2^) indicate that the bicelles spread to form a compact, insulating membrane. A negative potential shift is accompanied by a gradual decrease in the tilt of the acyl chains and thus an increase in the membrane thickness. Straightening of the acyl chains results in a decrease in the average area per hydrocarbon chain region in the DMPC bilayer. On the one hand, this transition in the DMPC bilayer causes a reorientation of the polar head groups, as observed in LB-LS DMPC bilayers.^88^ On the other hand, the space gained during the chain reorientation may allow for interaction, penetration of water and ions of the electrolyte solution into the polar headgroup region without significant changes in the polar headgroup orientation.

The positive-going potential scan displays a hysteresis in the changes of the tilt angle of the acyl chains in the DMPC floating bilayer (Figure 7). The average tilt angle remains close to 25° versus surface normal at *E*_m_ < 0 V, indicating that at negative membrane potentials a more vertical orientation of the acyl chains is stabilized. A ca. 10° increase in the tilt of the acyl chains is observed at the transition to *E*_m_ > 0 V (Figure 7).

Figure 8 shows PM IRRA spectra of the floating DMPC bilayer in the ν­(C=O) mode region of the ester carbonyl group that joins the polar headgroup with the acyl chains region.

**Figure 8:**
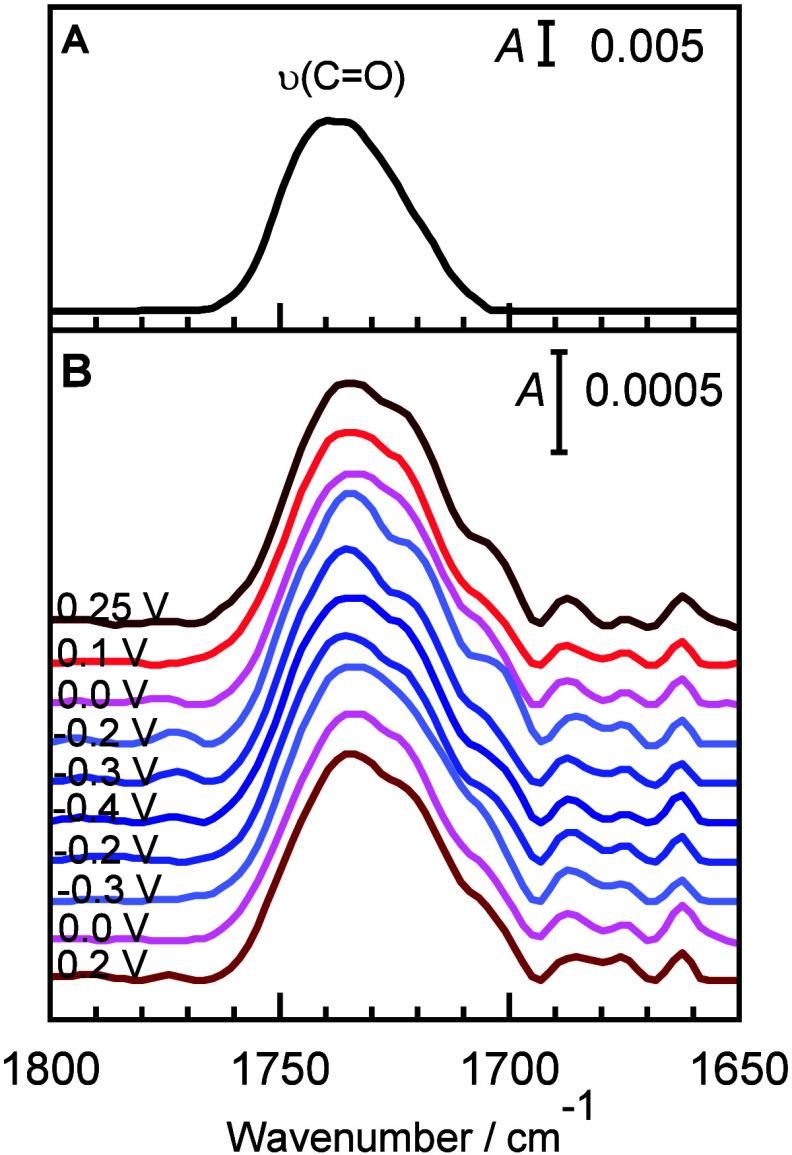
(A) PM IRRA spectrum of randomly distributed DMPC molecules in a bilayer thick film obtained from optical constants and (B) PM IRRA spectra of a floating DMPC bilayer obtained by spreading of bicelles on a β-Tg:SC_5_COOH monolayer adsorbed in a Au(111) electrode in 50 mM PBS in D_2_O (pD 7.6) at potentials marked in the figure in negative- and positive-going potential scans.

The asymmetric ν­(C=O) absorption band is composed of two overlapped IR absorption bands centered at ∼1740–1742 and ∼1722–1724 cm^–1^, originating from dehydrated and hydrogen-bonded hydrated ester carbonyl groups, respectively.^46,52,94^ A third weak absorption band appears at 1705 cm^–1^, which is primarily associated with the ν­(C=O) stretching mode in the SC_5_COOH in the spacer monolayer. The positions of the absorption maxima of the deconvoluted ν­(C=O) absorption are independent of the electrode potential.

To check if the orientation of the ester carbonyl groups responds to the electrode potential, the average tilt angle of the C=O bond (transition dipole vector of the ν­(C=O) mode) was calculated (Figure 9).

**Figure 9:**
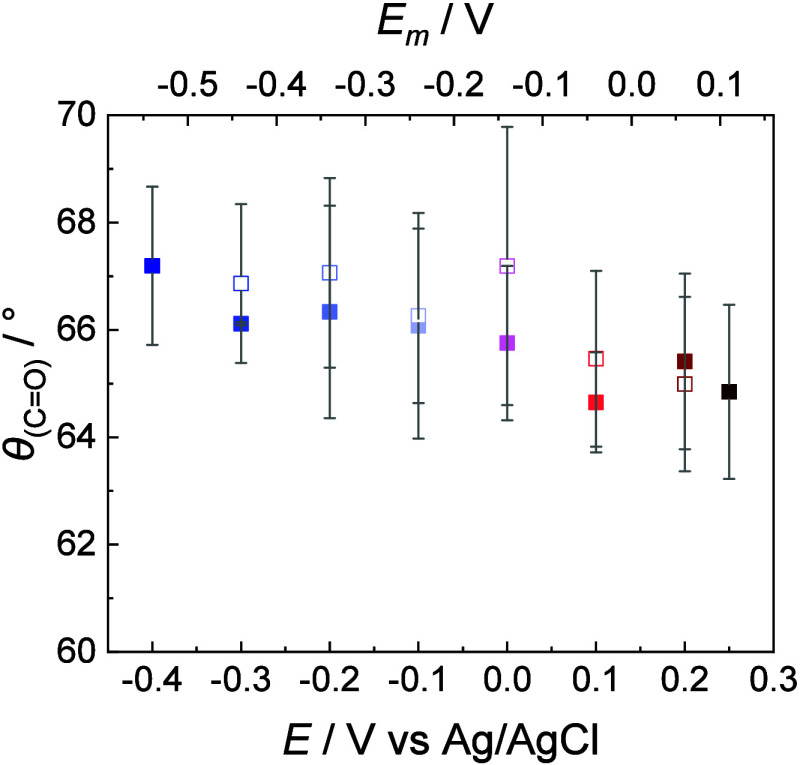
Tilt angle of the C=O ester carbonyl bonds in the DMPC floating bilayer deposited on the β-Tg:SC_5_COOH monolayer on a Au(111) electrode surface vs potential (membrane potential) plots. The electrolyte solution contained 50 mM phosphate buffer in D_2_O (pD 7.6): (filled squares) negative-going and (empty squares) positive-going potential scans. Colors of the points correspond to potential values at which PM IRRA spectra were recorded as shown in Figure 8.

The average tilt of the C=O bonds in the ester carbonyl group in the DMPC floating bilayer shifts linearly with potential from 67° at *E* = −0.4 V to 65° at *E* = 0.25 V, with a slope of ∼−4°/V. The ester carbonyl groups form an angle that is nearly perpendicular to the direction of the all-*trans* segments of the acyl chains.^88^ Thus, an increase in the tilt angle of the C=O bond at negative potentials implements the decrease in the tilt angle of the acyl chains. The slope of the C=O bond direction changes as a function of potential of ∼−4°/V and is much lower than the changes in the orientation of the acyl chains (18°/V). Thus, the potential driven changes in the tilt of the acyl chains are only partially compensated by the reorientation of the ester carbonyl and possibly the polar headgroup. Most likely, the network of hydrogen bonds and circulation of water in the polar headgroup region of the floating membrane is responsible for a rigid orientation of the ester carbonyl groups in the DMPC molecules.^91^

## Conclusions

Results of this work demonstrate that DMPC bicelles spread to form compact, defect-free, well-organized floating lipid bilayers. The electrochemical properties, ions and water transport through the membrane as well as membrane’s structure, at a molecular level, change in the vicinity of the *E*_pzfc_. The *E*_pzfc_ reflects the underlying interfacial characteristics of the membrane such as the dipole orientation (orientation of lipid and water molecules), direction of the electric field vector, and the surface composition, which themselves may influence the electrochemical behavior.

At negative membrane potentials, the *R*_m_ exhibits a linear dependence on potential. The nonohmic behavior of the lipid membrane indicates that changes in the *R*_m_ (conductance) may be driven by alterations in the membrane structure as well as interactions of the membrane with ions and water. Electrochemical impedance spectroscopy, quartz crystal microbalance, and infrared spectroscopy experiments clearly show that these two processes occur simultaneously. At the molecular scale, a decrease in the *R*_m_ at *E*_m_ < 0 V is associated with straightening of the acyl chains in DMPC molecules, which is accompanied by a much slower reorientation of the ester carbonyl groups. Quartz crystal microbalance experiments show an increase and decrease in frequency at negative membrane potentials (Figure 5). They are overlapped with an increase in membrane capacitance. This result indicates a small reduction and increase in the mass and suggests potential-driven out-flux and in-flux of water into the membrane vicinity. During the potential scan, the acyl chain assumes a more vertical orientation (tilt of 25° versus normal at *E*_m_ = −0.55 V), meaning that the membrane thickness increases and the average area per acyl chain in DMPC molecules decreases. A decrease in the area per acyl chains combined with a reorientation of the ester carbonyl groups provides space for ions and water to penetrate the polar headgroup of the membrane, enhancing the membrane conductance. Reversal of the potential scan inverses the observed processes until positive membrane potentials are reached. At *E*_m_ > 0 V, a formation of defects and an abrupt decrease in the *R*_m_ are observed. The tilt of the acyl chains increases by 10°, meaning that the area per chain increases. These sudden molecular-scale reorientations may lead to the formation of pores (defects) in an abrupt decrease in *R*_m_.

Our results confirm that bicelles spread to form a floating DMPC bilayer on the β-Tg:SC_5_COOH monolayer. This new method of the fabrication of lipid bilayer is superb when compared to spreading of vesicles. The spreading of vesicles depends strongly on the chemical nature of the substrate and its surface charge density. The spreading of vesicles may lead to adsorption of intact vesicles, formation of mutlibilayers or bilayer with large content of defects.^13,95,96^ For example, on the gold surface lipid vesicles spread to from a hemispherical film which slowly fuses to yield a single bilayer film.^96^ Transmembrane proteins can be incorporated into bicelles structure,^42,97,98^ providing native lipid environment to the proteins and stabilizing their structure compared to detergent-based micellar aggregates. Thus, bicelles filled with a transmembrane protein are ideal candidates for the fabrication of model membranes with incorporated membrane proteins.

## Supplementary Material



## Notes

Monitoring of the bicelles spreading by a quartz crystal microbalance (section S1), monitoring of the bicelles spreading by atomic force microscopy imaging (section S2), determination of the Young modulus from AFM measurements (section S3), determination of the potential of zero free charge of the floating DMPC bilayer obtained by bicelles spreading (section S4), results of the numeric analysis of the EIS data of the DMPC floating lipid bilayer spread from bicelles on a β-Tg:SC5COOH monolayer modified Au(111) electrode surface spread (section S5), quartz crystal microbalance with electrochemical control (section S6), and deconvolution of the PM IRRA spectra of the DMPC floating bilayer on the gold surface (section S7) (PDF)
