# Supplementary material for: Monitoring of Bicelles Spreading into Floating Lipid Bilayers
Source: Langmuir. 2025 Jul 30;41(31):20942–53. doi: 10.1021/acs.langmuir.5c02620 (PMC12356076; doi:10.1021/acs.langmuir.5c02620)
Supplement: Supplementary file 1 [file la5c02620_si_001.pdf]

Monitoring of bicelles spreading into floating lipid bilayers

SUPPORTING INFORMATION

*Justyna Bożek[1], Damian Dziubak[2], Arkadiusz Grempka[2], Slawomir Sek[2] and Izabella Brand[1*3]*

[1] Carl von Ossietzky Universität Oldenburg, Institute of Chemistry, 26111 Oldenburg, Germany

[2] University of Warsaw, Faculty of Chemistry, Biological and Chemical Research Centre, Zwirki i Wigury 101, Warsaw, 02-089, Poland

[3] Research Center for Neurosensory Sciences, Carl von Ossietzky Universität Oldenburg, D-26111 Oldenburg, Germany

Contents

S1. Monitoring of the bicelles spreading by quartz crystal microbalance S2

S2. Monitoring of the bicelles spreading by atomic force microscopy imaging S3

S3. Determination of the Young modulus from AFM measurements S4

S4. Determination of the potential of zero free charge of the floating DMPC bilayer obtained by bicelles spreading S5

S5. Results of the numeric analysis of the EIS data of the DMPC floating lipid bilayer sperad from bicleles on a *β*-Tg:SC_5COOH monolayer modified Au(111) electrode surface spread .S6

S6. Quartz crystal microbalance with electrochemical control S7

S7. Deconvolution of the PM IRRA spectra of the DMPC floating bilayer on the gold surface S8

References S9

S1. Monitoring of the bicelles spreading by quartz crystal microbalance

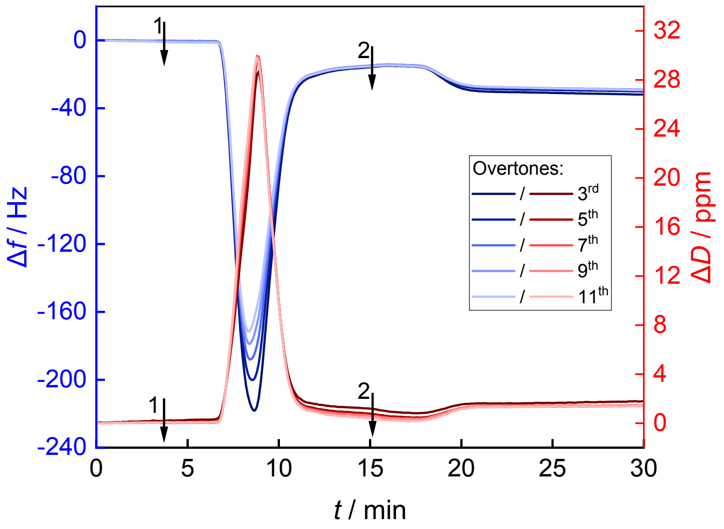

Figure S1. Changes in the frequency and dissipation energy versus time for spreading of DMPC/DHPC bicelles (q = 0.5) on a β-Tg:SC5COOH modified gold surface, 1: injection of the bicelles solution, 2.5 mM bicelles in 50 mM phosphate buffer in H_2O, pH 7.2; 2: washing of the surface with 50 mM phosphate buffer in H_2O, pH 7.2.

S2. Monitoring of the bicelles spreading by atomic force microscopy imaging

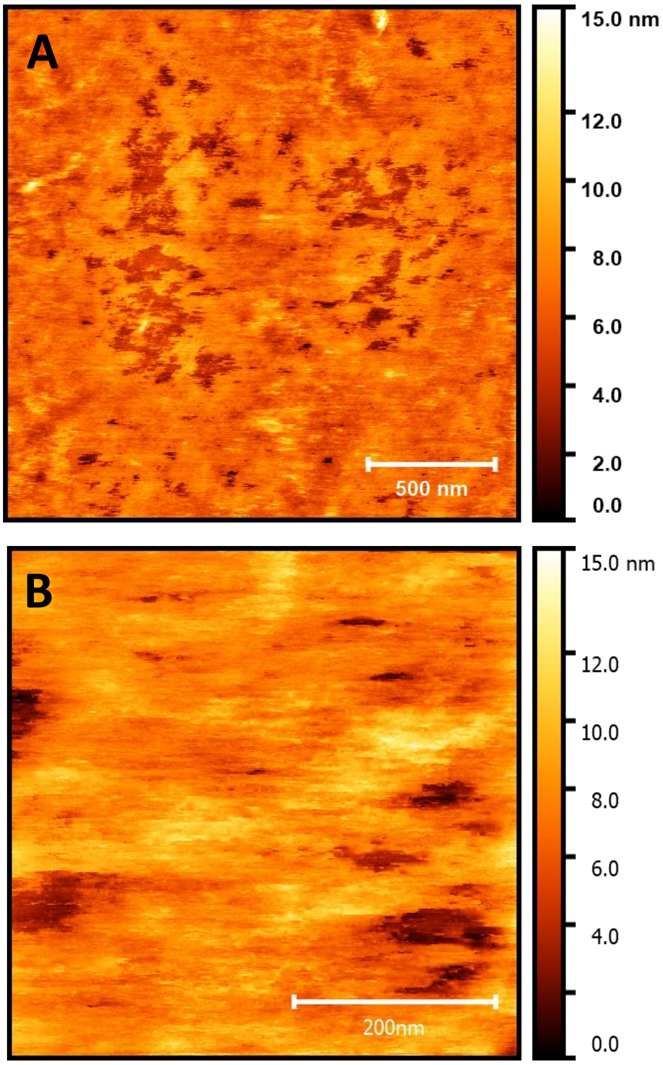

Figure S2. PeakForce QNM® mode AFM topography images of the floating DMPC bilayer obtained by the spreading of the bicelles from 50 mM phosphate buffer in H_2O, pH 7.2, on a *β*-Tg: SC_5COOH monolayer modified Au(111) surface at 25 °C. Image size is A. 2 × 2 µm, µm and B: zoomed in image 500 × 500 nm, scale bars are shown in each figure.

S3. Determination of the Young modulus from AFM measurements

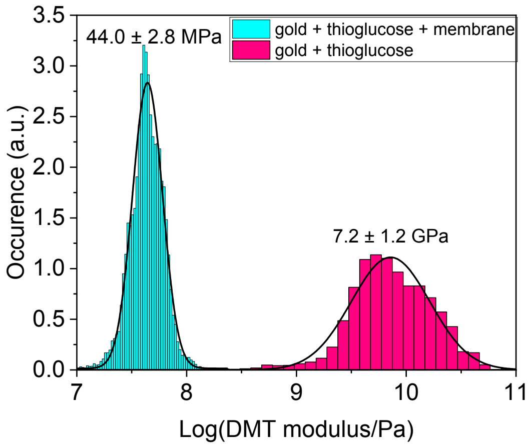

Figure S3. Histograms of the values of log(DMT modulus) obtained from quantitative nanomechanical mapping for the β-Tg:SC5COOH monolayer covered Au(111) surface and after the addition of the bicelle solution and spreading of a floating DMPC bilayer.

S4. Determination of the potential of zero free charge of the floating DMPC bilayer obtained by bicelles spreading

The potential of zero free charge (E_{pzfc}) was determined using the immersion method.[1] First, the electrode was suspended above the solution to which the potential was applied by using the chronoamperometric method. Then, the electrode was slowly immersed in the solution, and the current was measured over time. The resulting peak was integrated, and the charge density versus potential was plotted. The points were collected from 0.4 V to 0.0 V, with a step of –0.05 V. The intersection with the Y axis determined the E_{pfzc}.

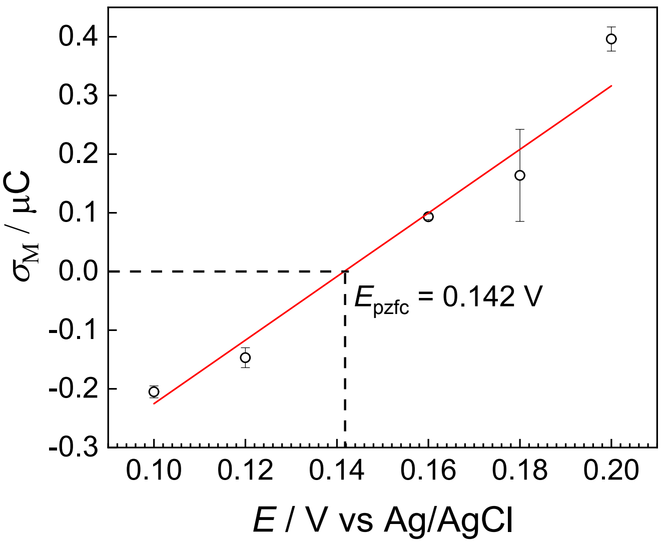

Figure S4. The potential of free zero charge of the DMPC floating bilayer spread by bicelles spreading on the surface of a *β*-Tg:SC5COOH monolayer covered Au(111).

S5. Results of the numeric analysis of the EIS data of the DMPC floating lipid bilayer sperad from bicleles on a *β*-Tg:SC_5COOH monolayer modified Au(111) electrode surface spread

Table S1. Results of the numeric analysis of the EIS data: *E* potential applied to the working electrode, Q_m – constant phase element of the membrane, R_m – resistance of the membrane, of the DMPC floating bilayer spread from bicelles on a *β*-Tg:SC5COOH monolayer modified Au(111) electrde in 50 mM phospahte buffer, pH 7.2. SD: standard deviation

**E / V vs Ag|AgCl**	**Q_m/ µF cm^{-2} s$^{\alpha-1}$**	**Q_m SD**	**α_m**	**R_m/ kΩ cm^2**	**R_m SD**
0.1	13.5	1.4	0.96	804	80
0	13.5	1.4	0.96	865	87
-0.2	15.9	1.6	0.96	908	338
-0.4	20.2	2.0	0.96	441	154
-0.2	15.3	1.5	0.97	659	66
0.1	11.6	1.2	0.96	1749	175
0.2	15.2	1.5	0.92	114	11

S6. Quartz crystal microbalance with electrochemical control

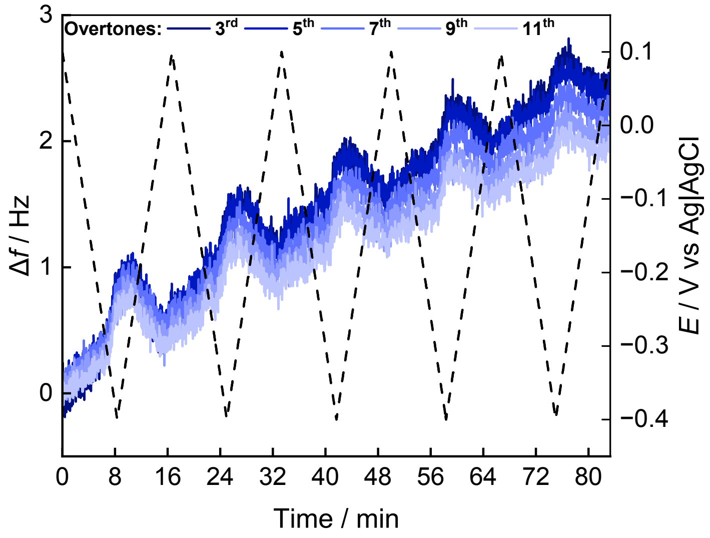

Figure S5. Overtones of frequency of a QCBM with an Au chip with a floating DMPC bilayer deposited by spreading of bicelles on a *β*-Tg:SC_5COOH monolayer and changes in the potential applied to the Au electrode as a function of time recorded during the first negative and following positive and negative potential scans. The potential scan rate was 1 mV s^{-1}. The electrolyte solution contained 50 mM phosphate buffer in H_2O, pH 7.2.

S7. Deconvolution of the PM IRRA spectra of the DMPC floating bilayer on the gold surface

The PM IRRA spectra of the floating DMPC bilayer were deconvoluted. The deconvolution procedure was done based on the positions of the minima of the second derivative of the PM IRRA spectra. In the deconvolution procedure, the wavenumbers of the absorption maxima were fixed, and the peak shape was fitted with a Gaussian curve. Figure S6 shows the deconvoluted spectra in the CH stretching IR absorption modes region of the DMPC floating bilayer spread from bicelles onto a *β*-Tg:SC5COOH monolayer modified Au(111) electrode.

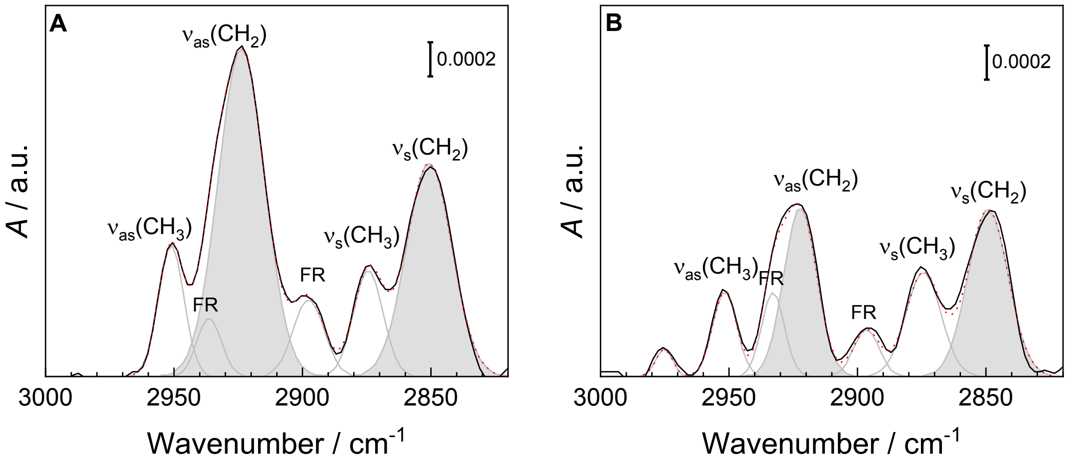

Figure S6. Deconvoluted *in situ* PM IRRA spectra in the CH stretching IR absorption modes region of the DMPC floating bilayer spread form bicelles onto a *β*-Tg:SC5COOH monolayer modified Au(111) electrode recorded at A: E = 0.25 V, and **B**: E = -0.4 V. **C**: Electrolyte solution contained 50 mM phosphate buffer in D_2O, pD = 7.6. Shaded IR absorption bands are quantitatively analyzed.

Figure S7 shows the deconvoluted spectra in the C=O stretching IR absorption modes region.

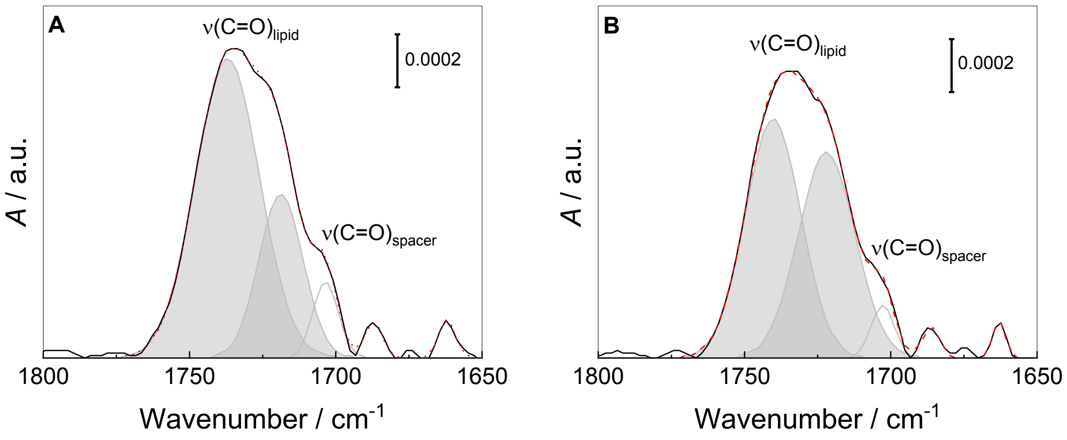

Figure S7. Deconvoluted *in situ* PM IRRA spectra in the C=O stretching IR absorption modes region of the DMPC floating bilayer spread form bicelles onto a *β*-Tg:SC5COOH monolayer modified Au(111) electrode recorded at A: E = 0.25 V, and **B**: E = -0.4 V. **C**: Electrolyte solution contained 50 mM phosphate buffer in D_2O, pD = 7.6. Shaded IR absorption bands are quantitatively analyzed.

Two molecules from the floating membrane environment contribute to the spectra shown in Fig. S7. The ester carbonyl groups are present in the DMPC lipids and give two strong IR absorption modes (1745-1715 cm^{-1} region). The carboxylic acid in the mercaptohexanoic acid present in the spacer region gives a weak IR absorption band ~1705 cm^{-1}.

References

(1) Hamm, U. W.; Kramer, D.; Zhai, R. S.; Kolb, D. M. The Pzc of Au(111) and Pt(111) in a Perchloric Acid Solution: An Ex Situ Approach to the Immersion Technique. *J. Electroanal. Chem.* **1996**, *414*, 85-89.
